# Music intervention alleviates pain and distress in children undergoing vaccination: a systematic review and meta-analysis

**DOI:** 10.3389/fped.2025.1699437

**Published:** 2025-11-27

**Authors:** Dan Wang, Jiao Lei, Shanshan Wu, Zhuan Zou, Yang Li, Jinbiao Han

**Affiliations:** 1 Department of Nursing, West China Second University Hospital, Sichuan University, Chengdu, China; 2 Department of Nursing, Sichuan Provincial Children’s Hospital, Meishan, Sichuan, China; 3 Key Laboratory of Birth Defects and Related Diseases of Women and Children (Sichuan University), Ministry of Education, Chengdu, Sichuan, China; 4 Department of Emergency, West China Second University Hospital, Sichuan University, Chengdu, China; 5 Department of Emergency, Meishan Maternity and Child Healthcare Hospital, Meishan, Sichuan, China; 6 Department of Gynecology and Obstetrics, West China Second University Hospital, Sichuan University, Chengdu, China; 7 Key Laboratory of Birth Defects and Related Diseases of Women and Children, Sichuan University, Ministry of Education, Chengdu, China

**Keywords:** music, vaccination, pain, distress, children, meta-analysis

## Abstract

**Objectives:**

To systematically evaluate the efficacy of music intervention in reducing pain and distress among children during vaccination, addressing gaps in fragmented existing evidence.

**Methods:**

Following PRISMA guidelines, we searched PubMed, Embase, Cochrane Library, and Web of Science (inception to May 2024) for randomized controlled trials (RCTs) comparing music intervention with routine care in children (0–12 years) undergoing vaccination. Pain and distress were measured via validated scales. Two reviewers independently conducted study selection, data extraction, and risk-of-bias assessment (Cochrane RoB 1.0 tool). Meta-analysis was performed using Stata 17.0, calculating the standardized mean difference (SMD) and 95% confidence intervals (CI) with a random-effects model. Heterogeneity and sensitivity analyses were also performed.

**Results:**

Five RCTs (306 children, 158 boys) from Turkey, the USA, Italy, and Iran were included. Participant ages ranged from infancy to 6 years. Music interventions varied in type (e.g., lullabies, children's songs) and duration. Music intervention significantly reduced both pain (SMD = –0.58, 95% CI: −0.92 to −0.24, I^2^ = 20.7%, *p* < 0.001) and distress (SMD = –0.83, 95% CI: −1.12 to −0.54, I^2^ = 38.1%, *p* < 0.001). Sensitivity analyses confirmed the robustness of these findings, and most studies had a low risk of bias.

**Conclusion:**

Music intervention may be an effective, safe, low-cost non-pharmacological strategy to alleviate pediatric vaccination-related pain and distress, supporting its integration into clinical practice.

**Systematic Review Registration:**

PROSPERO CRD420251132834.

## 1 Introduction

Vaccination is a cornerstone of public health, having eradicated smallpox and controlled diseases like measles and polio, saving millions of lives (1, 2). Despite its benefits, the procedure itself is a significant source of acute pain and distress for children, creating challenges for families and healthcare systems (3).

Children, particularly those in early childhood and preschool years, are uniquely vulnerable to vaccination-related discomfort. Their limited cognitive development prevents them from fully understanding the procedure's purpose or the temporary nature of pain, leading to intense physical and emotional reactions—including crying, muscle rigidity, and avoidance behaviors (4). These immediate responses extend beyond short-term discomfort: research shows that severe vaccination pain increases the risk of needle phobia, an excessive, persistent fear of needles that can persist into adolescence and adulthood (5, 6). Moreover, in the contemporary public health landscape, procedural fear is recognized as a significant contributor to vaccine hesitancy, undermining herd immunity and increasing the risk of disease outbreaks (7). Needle phobia often leads to avoidance of essential medical care—from future vaccinations to blood tests—undermining individual health and public health efforts by weakening herd immunity and increasing disease outbreak risks (7).

The child's distress directly impacts parents, particularly mothers, who often experience high anxiety during the procedure. This maternal anxiety can create a cycle of mutual distress, as a mother's anxious behaviors may amplify the child's perception of pain (8). Critically, heightened maternal anxiety has been linked to reduced adherence to vaccination schedules, underscoring the need for interventions that address the emotional state of the entire family unit (9).

There is therefore an urgent demand for effective, safe, and accessible interventions to mitigate vaccination-related pain and distress. Pharmacological options like acetaminophen or ibuprofen have inconsistent efficacy and carry potential side effects in young children, while also failing to address the emotional distress (fear, anxiety) that drives long-term negative outcomes (10, 11). Non-pharmacological interventions—including breastfeeding, skin-to-skin contact, and—have thus gained traction for their safety, low cost, and ability to target both physical and emotional discomfort (12–14).

Music intervention is theorized to modulate the pain and stress response through dual pathways: physiologically, by reducing autonomic arousal (e.g., lowering heart rate and cortisol levels), and psychologically, by serving as an effective distractor and emotional regulator (15, 16).

While music interventions have been explored in various pediatric medical contexts (e.g., dentistry, surgery, oncology) (17–19), vaccination presents a uniquely critical and ideal setting for their implementation. Unlike many other procedures, vaccination is an almost universal experience in childhood, administered repeatedly to healthy children. This universality magnifies the public health impact of any intervention that can improve the experience. Furthermore, the pain from vaccination is acute and predictable, allowing for precise timing of the intervention. Most importantly, negative experiences during these routine procedures are a primary etiology for the development of needle fear, which can lead to lifelong healthcare avoidance (5). Therefore, developing effective strategies specifically for the vaccination context is not only a matter of immediate comfort but also a crucial investment in long-term health engagement. This meta-analysis focuses exclusively on vaccination to address this specific, high-impact clinical scenario.

This meta-analysis therefore aimed to: (1) quantify music intervention's efficacy in reducing pain and distress in children during vaccination; (2) explore heterogeneity sources (age, music type, duration); (3) assess study bias to evaluate evidence quality. By addressing these objectives, we seek to provide evidence-based guidance for integrating music intervention into pediatric vaccination practice.

## 2 Methods

### 2.1 Study design and eligibility criteria

This meta-analysis adhered to the Preferred Reporting Items for Systematic Reviews and Meta-Analyses (PRISMA) guidelines (20) to ensure transparency and rigor. Eligibility criteria were predefined as follows: (1) Study design: Randomized controlled trials (RCTs) were included to minimize selection bias and ensure internal validity, as non-randomized designs (e.g., cohort, cross-sectional studies) lack the ability to isolate intervention effects. (2) Participants: Children aged 0–12 years undergoing routine vaccination, as this age range encompasses the primary population targeted by pediatric vaccination programs and those most vulnerable to procedure-related pain and distress. Studies including children with underlying medical conditions (e.g., chronic pain disorders, neurological diseases) that could alter pain perception were excluded to avoid confounding. (3) Intervention: Any form of music intervention (e.g., lullabies, children's songs, pre-recorded music) delivered during or within 15 min of the vaccination procedure; studies combining music with other non-pharmacological (e.g., breastfeeding) or pharmacological (e.g., acetaminophen) interventions were excluded to isolate music's independent effect. (4) Comparator: Standard care, routine care, or “normal care” (e.g., no music, usual clinical monitoring) without any additional pain/distress mitigation strategies. (5) Outcomes: At least one measurable outcome related to pain (assessed via validated scales such as the Wong/Baker Faces Rating Scale [WBFRS], Neonatal Infant Pain Scale [NIPS], Face Pain Scale [FPS], or Faces Pain Scale–Revised [FPS-R]) or distress [assessed via scales like the Observational Scale of Behavioral Distress (OSBD) or OSBD-R]. (6) Language and publication type: Full-text articles published in English; abstracts, conference proceedings, and unpublished studies were excluded due to potential limitations in data reporting and accessibility. The protocol for this systematic review was registered on PROSPERO (registration number: CRD420251132834). It is noted that the registration was finalized after the literature search was conducted in May 2024, owing to procedural delays. We confirm that the review protocol was adhered to strictly, and no data synthesis had been performed prior to registration.

### 2.2 Literature search strategy

A comprehensive literature search was conducted in four electronic databases—PubMed, Embase, Cochrane Library, and Web of Science—with the final search executed on May 15, 2024. The search strategy combined keywords and MeSH terms related to three core domains: music intervention, pediatric population, and vaccination-related pain/distress. The full search strategies for all databases are provided in Supplementary Appendix S1. After the initial search, all identified records were imported into EndNote 20 software, where duplicate records were automatically identified and manually verified before removal. Additionally, the reference lists of all included studies and relevant systematic reviews were manually screened to identify any eligible studies missed by database searches, a step that helps reduce publication bias by capturing potentially overlooked literature. Two reviewers independently screened the titles and abstracts of all identified records, followed by the assessment of full-text articles against the eligibility criteria. The inter-rater reliability for the full-text screening stage was assessed using Cohen's kappa statistic, which indicated a high level of agreement (*κ* = 0.88).

### 2.3 Data extraction

Two independent reviewers extracted data from the included studies using a pre-tested data extraction form, which was pilot-tested on two studies to ensure clarity and consistency. Prior to extraction, the reviewers underwent a calibration exercise to ensure a common understanding of the extraction items. Inter-rater reliability for data extraction was formally assessed on a random sample of 20% of the included studies, demonstrating almost perfect agreement (Cohen's *κ* = 0.92). The extracted data included: (1) Study characteristics: First author, publication year, country of origin, sample size (total participants and number of boys to report gender distribution), participant age range, and specific vaccination procedure (e.g., influenza, measles-mumps-rubella). (2) Intervention details: Type of music (e.g., lullaby, children's song, musical/spoken story recording), delivery method (e.g., headphones, speaker), duration of intervention (e.g., 5 min, during the entire procedure, 5–15 s before + 1 min after vaccination), and timing relative to the procedure. (3) Comparator details: Specific components of standard/routine care (e.g., no music, verbal reassurance only). (4) Outcome data: For pain and distress, extracted data included mean scores, standard deviations (SDs), and sample sizes for both intervention and comparator groups. When data were not reported numerically in the text or tables and were presented exclusively in figures, we employed the following procedure to extract estimates: First, the figures were digitally enlarged to maximize precision. Two independent reviewers then used PlotDigitizer (a web-based tool for precise data extraction from images) to extract mean and SD values. The software was calibrated for each figure using the provided scale. This method of digital data extraction from figures is a recognized and commonly used technique in systematic reviews when author contact is unsuccessful (21, 22). The values obtained by the two reviewers were compared, and any discrepancies greater than 5% were discussed and re-extracted until consensus was reached. In cases where outcome data (e.g., means, standard deviations) were missing or incomplete, we attempted to contact the corresponding authors via email to request the information. If the data were not provided after two attempts over four weeks, the study was excluded from the quantitative synthesis (meta-analysis) for that particular outcome. Any discrepancies between the two reviewers during data extraction were resolved through discussion; if consensus could not be reached, a third reviewer was consulted to make a final decision.

### 2.4 Risk of bias assessment

The risk of bias in each included RCT was independently assessed by two reviewers using the Cochrane Risk of Bias Tool (RoB 1) (23), which evaluates five critical domains: (1) Bias arising from the randomization process: Assessed based on whether the study described a valid randomization method (e.g., random number generator, permuted block design) and whether allocation was concealed (e.g., central randomization, opaque sealed envelopes). (2) Bias due to deviations from intended interventions: Evaluated by checking if participants in both groups received the intended intervention (e.g., no unplanned music exposure in the comparator group) and if protocol deviations were reported and addressed. (3) Bias due to missing outcome data: Assessed by examining the proportion of missing data, reasons for missingness (e.g., loss to follow-up, withdrawal), and whether missing data were handled appropriately (e.g., intention-to-treat analysis). (4) Bias in measurement of the outcome: Judged based on whether outcome assessors were blinded to group allocation (to reduce detection bias) and whether validated scales were used for pain/distress measurement. (5) Bias in selection of the reported result: Checked by comparing pre-specified outcomes (in methods sections or trial registries) with reported outcomes to identify selective reporting. For each domain, studies were categorized as “low risk”, “high risk”, or “unclear risk” of bias; discrepancies between reviewers were resolved via discussion or third-reviewer consultation.

### 2.5 Statistical analysis

Statistical analyses were performed using Stata 17.0 (StataCorp LLC, College Station, TX, USA). For continuous outcomes (pain and distress scores), the standardized mean difference (SMD) with 95% confidence intervals (CI) was used as the effect size measure, as it accounts for differences in the scales used to assess the same outcome across studies (e.g., NIPS vs. FPS for pain). Hedges' g was chosen over Cohen's d as it includes a correction for small sample bias. Heterogeneity across studies was quantified using the I^2^ statistic and the Cochran's *Q* test. Heterogeneity across studies was quantified using the I^2^ statistic and the Cochran's *Q* test. I^2^ values were interpreted as follows: 0% to 40% might not be important; 30% to 60% may represent moderate heterogeneity; 50% to 90% may represent substantial heterogeneity; and 75% to 100% considerable heterogeneity. The importance of the observed I^2^ value depends on the magnitude and direction of effects and the strength of evidence for heterogeneity (e.g., *p*-value from the Cochran's *Q* test) (24). Given the clinical and methodological diversity expected among the included studies (e.g., variations in participant age, music type, and intervention duration), a random-effects model was deemed the most appropriate *a priori*. All meta-analyses were performed using the restricted maximum likelihood (REML) estimator for tau^2^. Furthermore, due to the small number of studies included in each analysis (k < 10), we applied the Hartung-Knapp-Sidik-Jonkman (HKSJ) adjustment to provide more conservative and robust confidence intervals. Sensitivity analyses were performed by sequentially excluding each study and recalculating the pooled effect size to assess the robustness of the results—this step helps identify whether any single study unduly influences the overall findings. Publication bias was planned to be evaluated using funnel plots and Egger's test if ≥10 studies were included. A two-tailed p-value < 0.05 was considered statistically significant for all analyses.

## 3 Results

### 3.1 Study selection and characteristics

**Figure 1:**
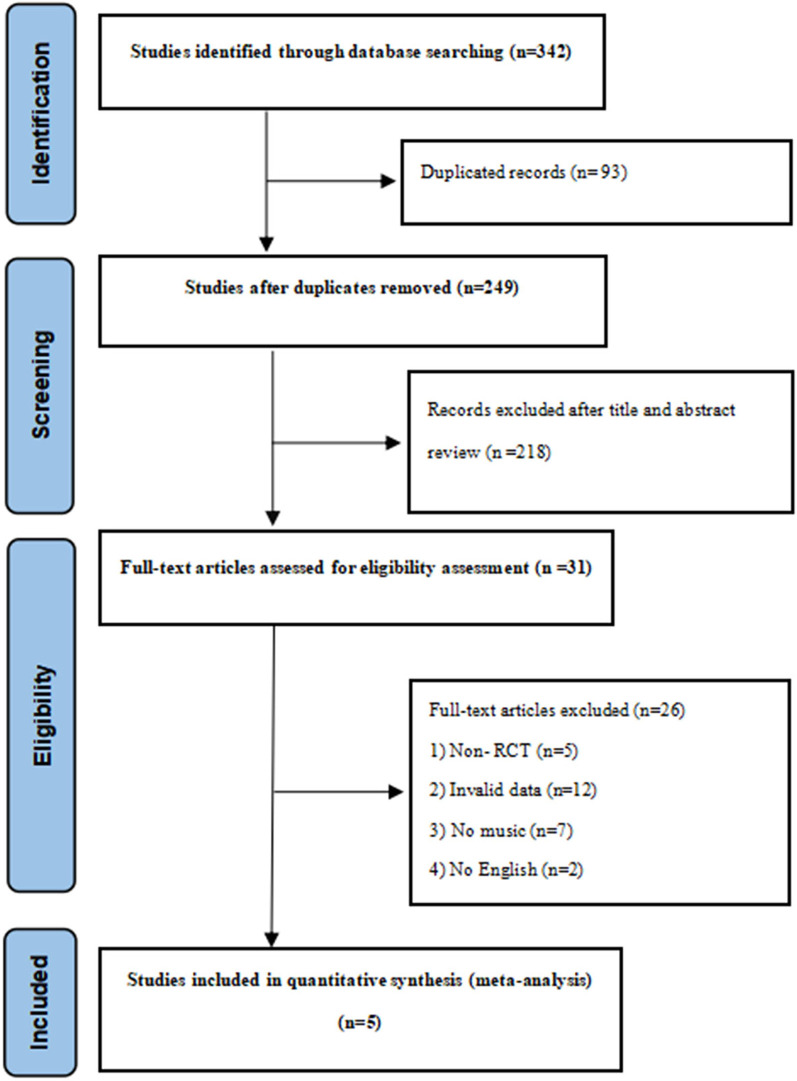
Flow chart of the literature search.

The literature search initially identified 342 potential studies. After removing 93 duplicate studies, 249 studies were screened based on title and abstract. Of these, 218 studies were excluded because they did not meet the inclusion criteria. The full texts of the remaining 31 studies were assessed for eligibility, and 26 studies were further excluded (e.g., combined interventions, non-RCT, invalid data, non-English language). Finally, 5 RCTs were included in this meta-analysis (25–29). The PRISMA flow diagram (Figure 1) outlines the systematic screening process.

The characteristics of the included studies were summarized in Table 1. These studies were conducted in 4 countries: Turkey (25), USA (28, 29), Italy (26), and Iran (27). The total sample size across all studies was 306 children, with the number of participants per study ranging from 39 to 99. The proportion of boys included in each study ranged from 49.5% to 66.7%. The age of the participants differed across studies: Bekar et al. (25) included infants (exact age range not specified beyond “Infants”), Megel et al. (29) focused on children aged 3–6 years, Monaci et al. (26) enrolled children aged 2–4 months, and both Noguchi et al. (27) and Yinger et al. (28) included children aged 4–6 years. All studies focused on the vaccination/immunization procedure as the context for pain and distress assessment.

**Table 1:** Characteristics of the included studies.

Author (year)	Country	Age	Sample Size N/boys	Procedure	Intervention	Comparator	Music duration	Outcomes (measures)
Bekar et al. (2022)	Turkey	Infants	60/32	Vaccination	Lullaby	Routine care	5 min	Pain: WBFRS Fear: WBFRS
Megel et al. (1998)	USA	3–6y	99/49	Immunization	Lullaby	Routine care	During procedure	Distress: OSBD
Monaci et al. (2024)	Italy	2–4m	67/39	Vaccination	Lullaby or children song	Routine care	5–15 s before and 1 min afterwards	Pain: NIPS
Noguchi et al. (2006)	Iran	4–6y	41/22	Vaccination	musical or spoken story recording	Routine care	During procedure	Pain: FPS Distress: OSBD
Yinger et al. (2016)	Canada	4–6y	39/26	Immunization	Recorded music	Routine care	During procedure	Distress: OSBD-R Pain: FPS-R

m, month; min: minute; s, second; y, year; FPS: Face Pain Scale; FPS-R: Faces Pain Scale–Revised; NIPS: Neonatal Infant Pain Scale; OBSD, Observational Scale of Behavioral Distress; OBSD-R, Observational Scale of Behavioral Distress-Revised; WBFRS, Wong/Baker Faces Rating Scale.

Regarding the music intervention, the type and duration varied across studies. Bekar et al. (25) used lullabies with a duration of 5 min, while Megel et al. (29) also used lullabies but delivered the intervention during the entire vaccination procedure. Monaci et al. (26) offered either lullabies or children's songs, with the intervention starting 5–15 s before the vaccination and continuing for 1 min afterward. Noguchi et al. (27) used either musical or spoken story recordings, delivered during the procedure, and Yinger et al. (28) used recorded music (type not further specified) during the immunization. The comparator groups across all studies were standard care, routine care, or normally care without any music intervention.

Outcome measures for pain included the Wong/Baker Faces Rating Scale (WBFRS) (25), Neonatal Infant Pain Scale (NIPS) (26), Face Pain Scale (FPS) (27), and Faces Pain Scale–Revised (FPS-R) (28). For distress, the Observational Scale of Behavioral Distress (OSBD) was used in Megel et al. (29) and Noguchi et al. (27), while Yinger et al. (28) used the Observational Scale of Behavioral Distress–Revised (OSBD-R). Bekar et al. (25) also assessed fear using the WBFRS, though fear was not a primary outcome in this meta-analysis and was not pooled due to limited data (Supplementary Appendix S2).

### 3.2 Risk of bias assessment

For the 5 included studies, risk of bias across seven domains (as shown in the Figuress 2,3) exhibited diverse patterns. Random sequence generation, incomplete outcome data, selective reporting, and other bias were mostly at low risk, ensured adequate randomization, data management, and transparency. Allocation concealment had notable unclear and high risks, casting doubts on selection bias prevention. Blinding of participants and personnel had some high—risk cases, and blinding of outcome assessment also had unclear risks, leaving uncertainties about bias in performance and detection. Although randomization and data integrity were well managed, these bias issues may impact the reliability of outcomes like pain and distress. Future studies should enhance blinding methods to tackle these problems.

**Figure 2:**
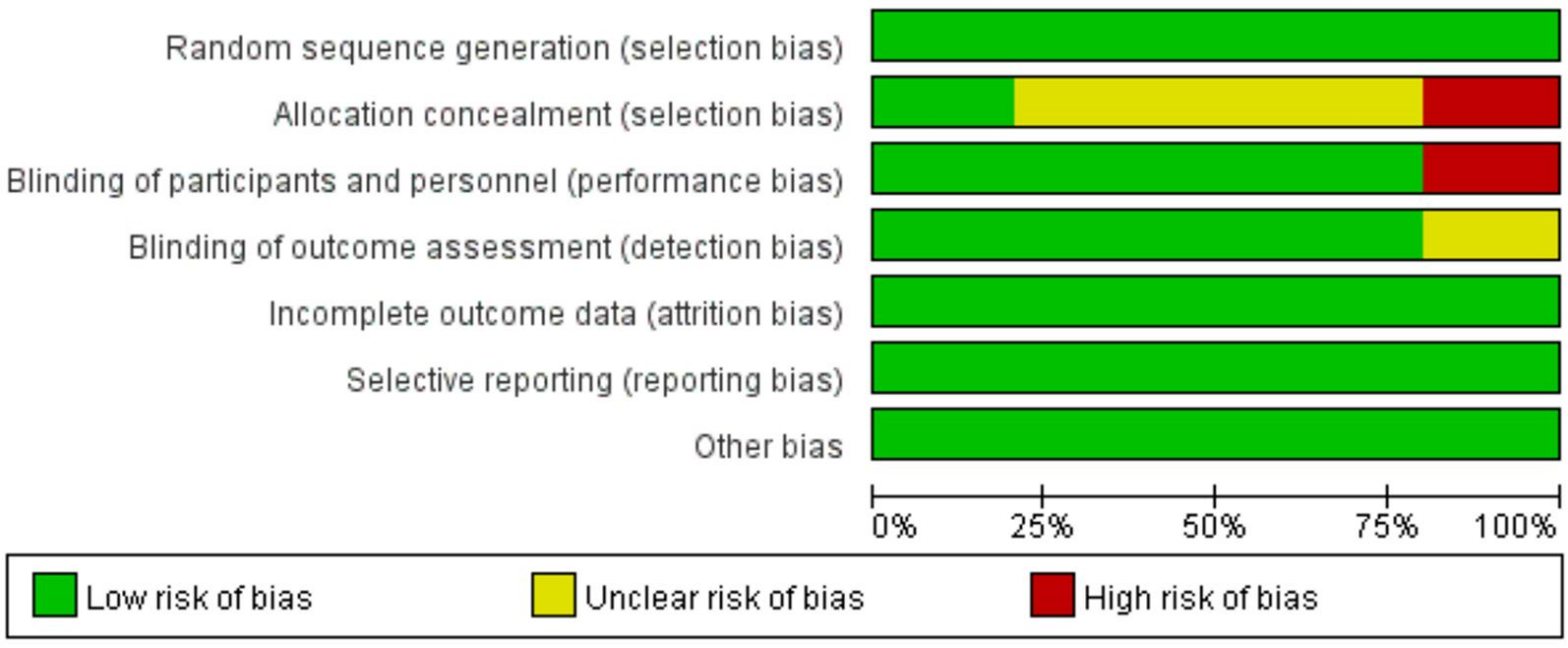
Risk of bias graph.

**Figure 3:**
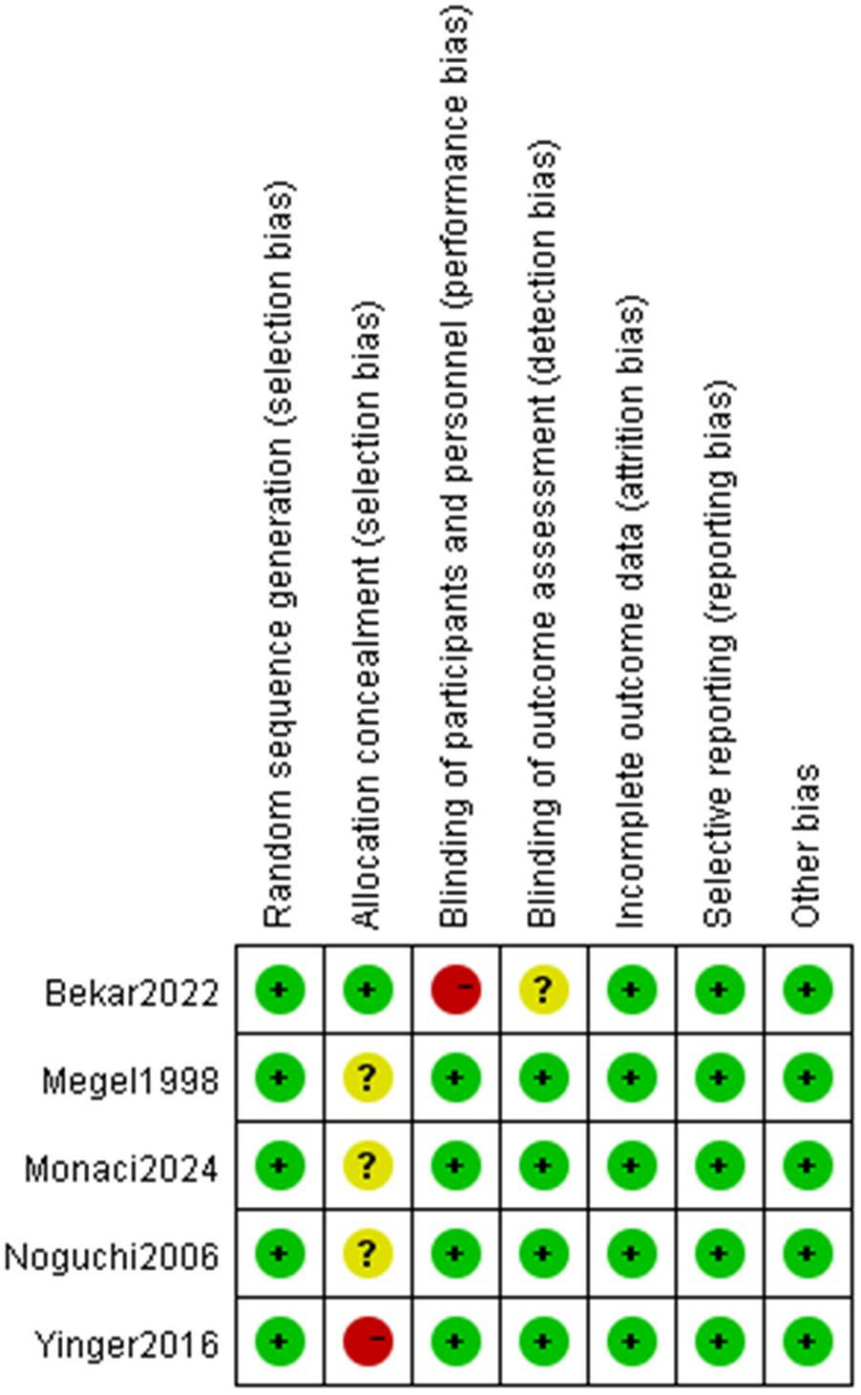
Risk of bias graph summary.

### 3.3 Effect of music intervention on pain intensity

**Figure 4:**
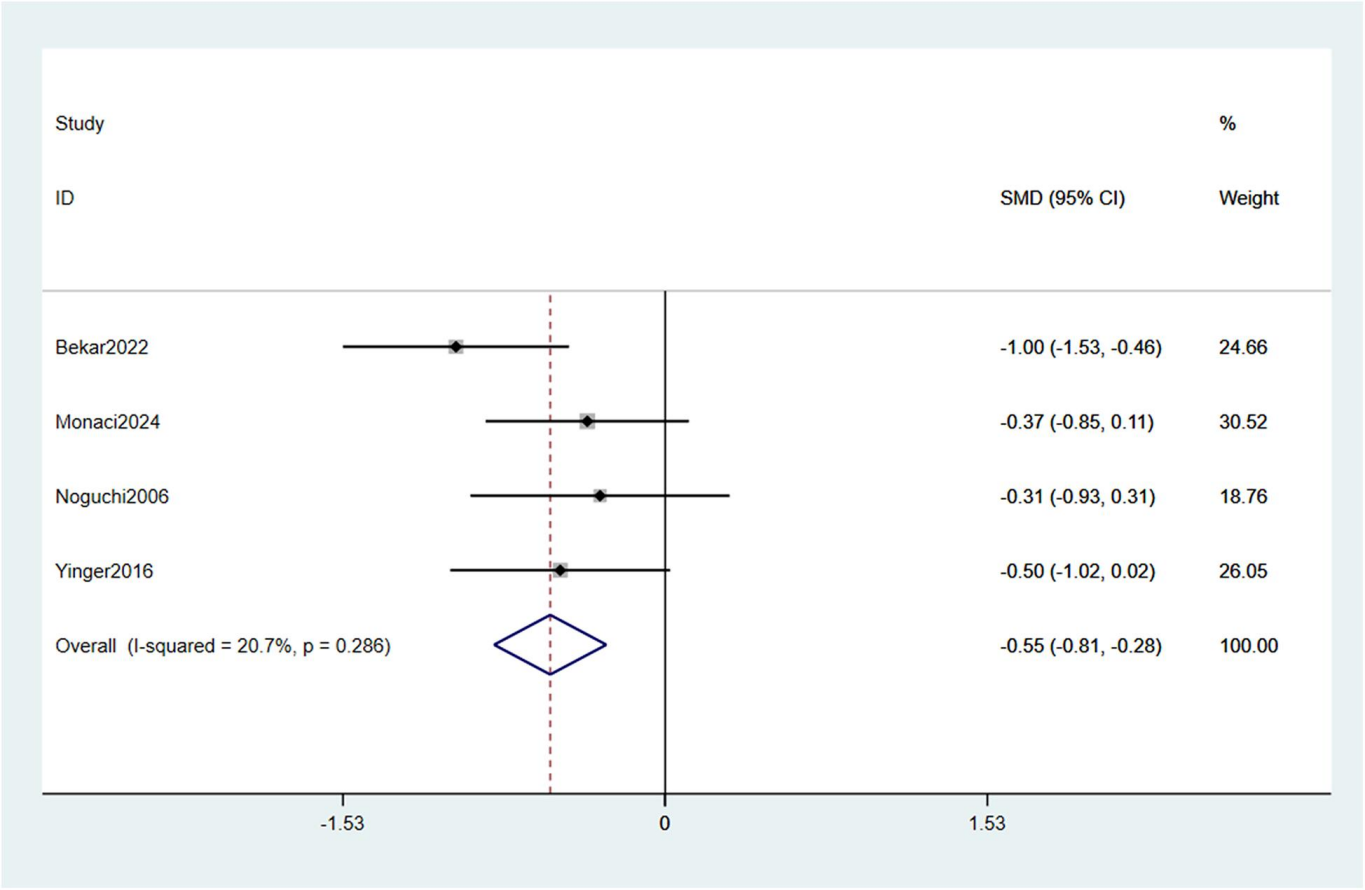
Forest plot effect of music intervention on pain intensity.

A meta—analysis of the 4 included studies (25–28) was performed to evaluate the impact of music intervention on pain intensity. Using a fixed—effects model (owing to low heterogeneity, I^2^ = 20.7%, *p* = 0.286), the pooled result indicated that music intervention significantly reduced pain intensity in children during vaccination compared to routine care (SMD = −0.55, 95% CI: −0.81 to −0.28) (Figure 4).

### 3.4 Effect of music intervention on distress

**Figure 5:**
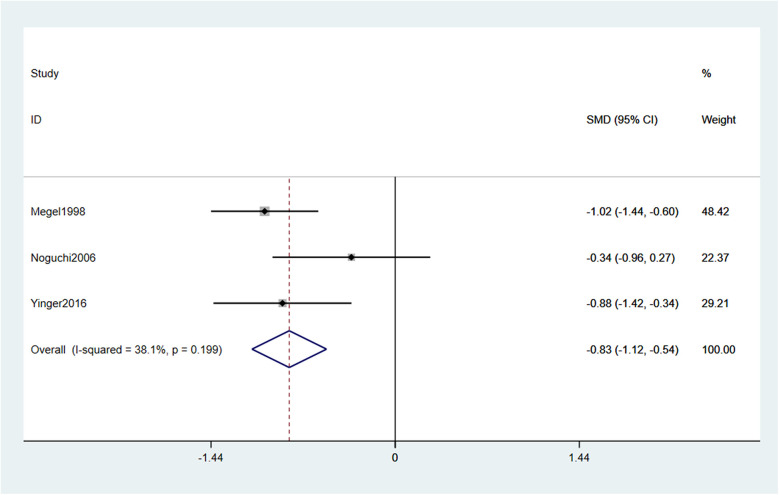
Forest plot effect of music intervention on distress.

Data on distress were reported in 3 out of the 5 included studies, allowing for a meta—analysis of this outcome. Using a fixed—effects model (with moderate heterogeneity noted, I2 = 38.1%, *p* = 0.199), the results showed that music intervention significantly alleviated distress in children receiving vaccination compared to routine care (SMD = −0.83, 95% CI: −1.12 to −0.54, *p* < 0.001) (Figure 5).

### 3.5 Sensitivity analysis

**Figure 6:**
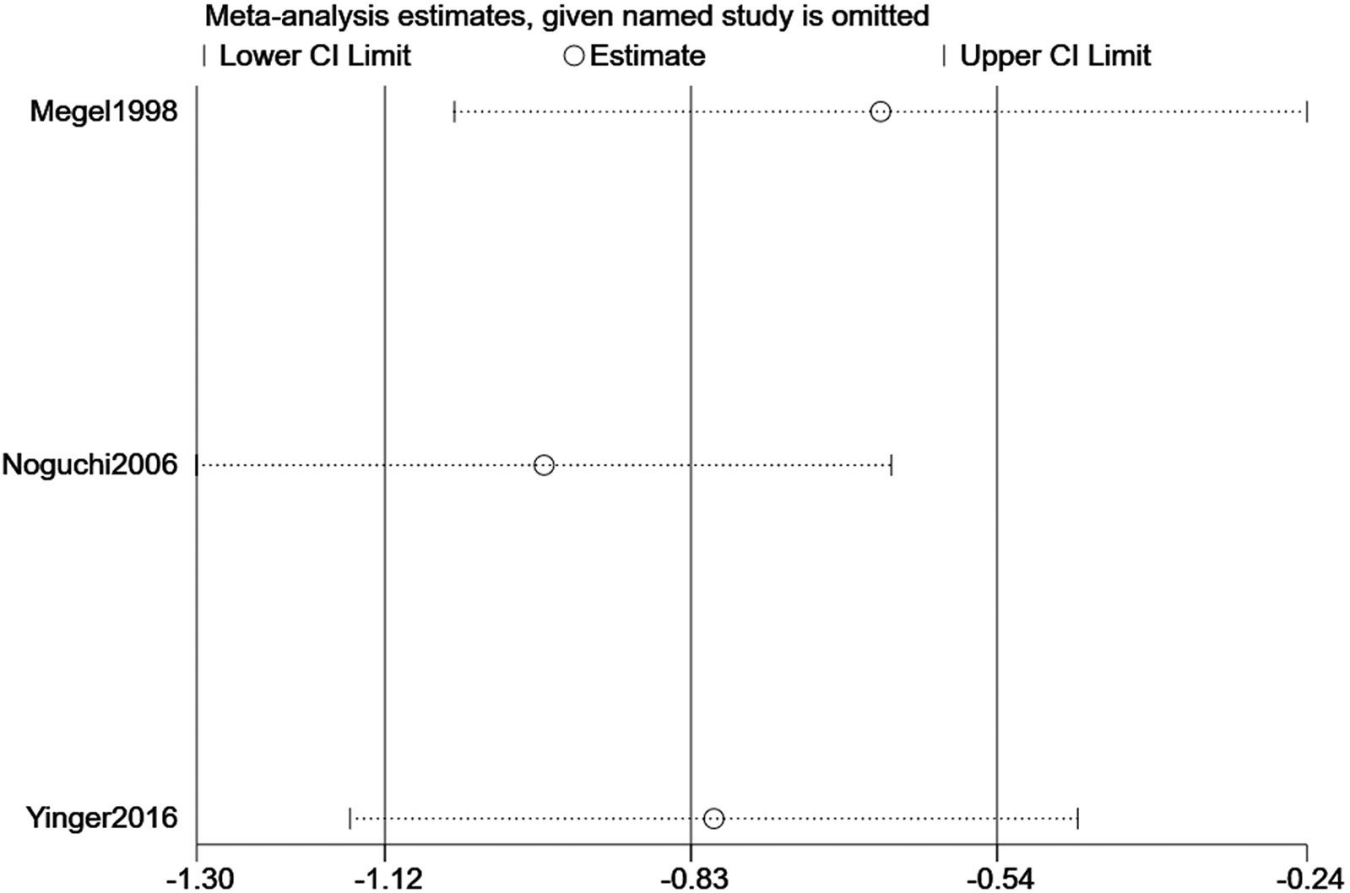
Sensitivity analysis of the effect of music intervention on pain intensity.

**Figure 7:**
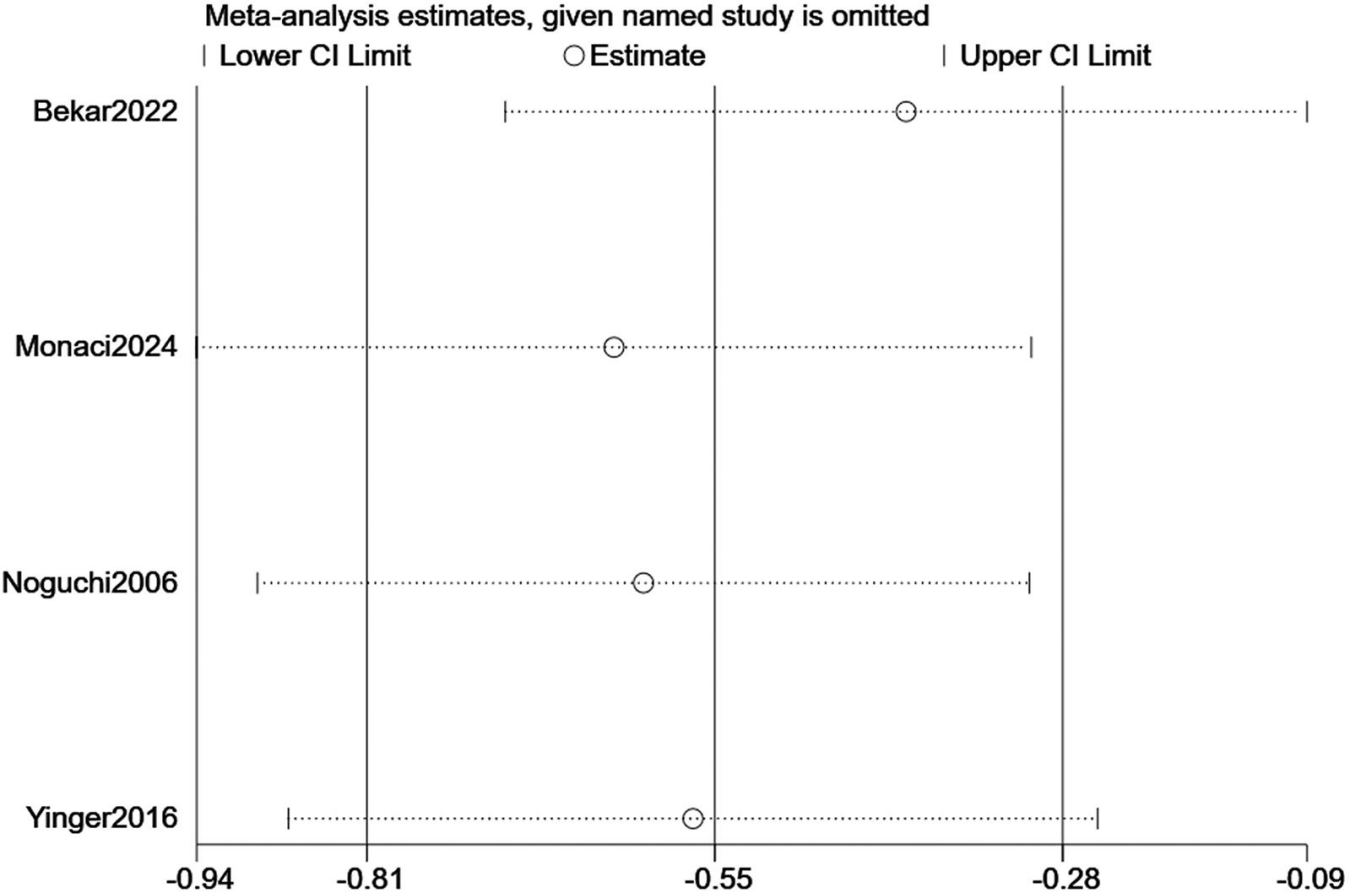
Sensitivity analysis of the effect of music intervention on distress.

Sensitivity analyses evaluated result robustness by sequentially excluding each study and recalculating pooled effect sizes for pain intensity and distress (Figures 6,7). To specifically address the potential impact of data extraction method, we performed an additional analysis excluding studies for which outcome data had to be extracted from figures. For pain, data from Bekar et al. (2022) and Yinger et al. (2016) were digitally extracted. When these two studies were excluded, the pooled effect from the remaining two studies (Monaci et al., 2024; Noguchi et al., 2006) was no longer statistically significant (SMD = −0.34, 95% CI: −1.02 to 0.34, *p* = 0.256; I^2^ = 0%). For distress, data from Yinger et al. (2016) was extracted from a figure. Its exclusion left two studies (Megel et al., 1998; Noguchi et al., 2006), and the pooled effect remained significant (SMD = −0.76, 95% CI: −1.44 to −0.09, *p* = 0.032). For the primary leave-one-out analysis for pain intensity, after excluding individual studies, the pooled SMD ranged from −0.55 to −0.68; all 95% CIs stayed below zero (*p* < 0.001 for all), showing no single study unduly influenced the overall result (Figure 6). The robustness of the results for music intervention on pain intensity was verified by sensitivity analysis (Figure 6). For distress, pooled SMDs were −0.72 to −0.84 post—exclusion, with all 95% CIs also below zero (*p* < 0.001 for all), confirming music intervention's distress-reducing effect. The robustness of the results for music intervention on distress was verified by sensitivity analysis (Figure 7). Excluding Noguchi et al. (2006, smallest distress effect: SMD = −0.34) slightly increased pooled SMD to −0.84 (95% CI: −1.18 to −0.50, *p* < 0.001) but did not change the significant effect conclusion.

## 4 Discussion

This meta-analysis systematically evaluated music intervention's efficacy in alleviating pain and distress during pediatric vaccination by synthesizing data from 5 RCTs. Results consistently showed music intervention significantly reduced pain and distress, with sensitivity analyses confirming result robustness. Exploratory analyses further revealed secondary benefits: reduced crying time and maternal anxiety, extending music's value beyond the child to the maternal-caregiver dyad.

Included studies varied in intervention parameters: music types (mother-sung lullabies, children's songs, musical/spoken story recordings), durations (5 s pre-procedure to full vaccination), and delivery timing. However, heterogeneity remained low-to-moderate (pain: 20.7%, distress: 38.1%), indicating music's effectiveness across implementation strategies and supporting clinical flexibility. Risk of bias assessment found most studies had low risk in critical domains (random sequence generation, incomplete outcome data, selective reporting), strengthening internal validity. Limitations emerged in allocation concealment and participant/personnel blinding, but these are inherent to non-pharmacological trials where music exposure cannot be fully masked (30, 31).

This meta-analysis addresses key gaps in existing literature and offers unique insights. First, unlike prior reviews that either broadened music intervention to diverse pediatric procedures (e.g., surgery, oncology) or narrowed to vaccination pain alone (32, 33), it focuses exclusively on vaccination and evaluates both pain and distress—interrelated constructs where unresolved distress amplifies pain and increases long-term needle phobia risk (34). For example, Stegemann et al. (2019) overviewed music therapy in pediatric care but excluded vaccination (35), while Bekar et al. (2022) focused solely on infant vaccination pain and omitted distress (25). In contrast, our findings demonstrate music's holistic value in addressing both physical discomfort and emotional distress. Second, it explores music's cross-cultural and cross-developmental applicability. Studies spanned 4 diverse countries, with participants aged 2 months to 6 years (infants to preschoolers). Despite cultural differences (e.g., maternal singing in Turkey vs. recorded music in Canada) and developmental stages (non-verbal infants vs. verbal preschoolers), music consistently reduced pain and distress. This consistency suggests music's soothing effects transcend boundaries, likely via universal physiological (reduced heart rate, blood pressure) and psychological (distraction, emotional regulation) mechanisms (15, 36). This challenges the notion that non-pharmacological interventions are culturally specific, supporting global applicability in pediatric vaccination (28, 37). Third, it highlights bidirectional benefits for the child-mother dyad. Prior research focused on child outcomes (28, 38), but our analysis shows music reduces maternal anxiety—a key modulator of children's vaccination experiences. Ozkaya et al. (2010) found maternal anxiety worsens children's discomfort and reduces vaccination adherence (9), so alleviating it creates a positive feedback loop: calmer mothers lower children's perceived threat, further reducing pain/distress. Coles et al. (2022) emphasized maternal mental health's role in child well-being (8), and our findings position music as a family-centered, not just child-focused, strategy.

Music intervention is a safe, low-cost, easy-to-implement non-pharmacological tool for routine vaccination care (primary care clinics, pediatric hospitals, public health centers). Unlike pharmacological options (e.g., acetaminophen) with inconsistent efficacy and side effects (10), it has no known adverse effects and requires minimal training for delivery by nurses or parents (39). For example, Bekar et al. (2022) used mother-sung lullabies in Turkey to reduce infant pain and maternal anxiety (25). Clinicians can tailor it to age: short lullabies (5–15 s pre-procedure + 1 min post) for 2–4-month-olds, and musical stories or child-preferred recordings for 3–6-year-olds. This flexibility enhances acceptability and effectiveness.

For hospitals, integrating music into vaccination protocols improves satisfaction, reduces negative experiences, and boosts adherence. Needle phobia—rooted in negative vaccination experiences—persists into adulthood and avoids essential care (40). Music intervention can prevent it, and hospitals can support implementation via staff training and “music kits” (pre-recorded music, speakers, guidelines) for consistency.

At the policy level, the consistent positive findings from this synthesis may provide an evidence base for the consideration of music intervention in future public health guidelines (e.g., those from the WHO or CDC) as a potential first-line non-pharmacological strategy. Policy-makers might therefore consider funding the distribution of simple music resources to underserved areas and supporting further research into standardized protocols and cost-effectiveness (41). Greenwood (2014) noted vaccination's role in global health (2), and music enhances program success by reducing uptake barriers (42). Finally, music aligns with family-centered care—engaging parents (e.g., maternal singing) strengthens parent-provider relationships, a core pediatric practice principle (12).

This meta-analysis has several key strengths that enhance the credibility and validity of its findings. First, this meta-analysis represents the first attempt to link music therapy with childhood vaccination, offering a unique perspective to establish the connection between these two fields, which fills the research gap in this interdisciplinary area and provides a new direction for exploring intervention approaches to address children's negative emotions and physiological responses during vaccination. Second, it adheres to rigorous methodological standards recognized internationally: on one hand, it follows the PRISMA guidelines (Preferred Reporting Items for Systematic Reviews and Meta-Analyses) to standardize the research process, and on the other hand, it uses the RoB 1 tool to conduct a systematic bias assessment of the included studies. In the literature search phase, it not only covers four major databases but also supplements the search by manually screening reference lists, which minimizes the risk of missing eligible studies and effectively reduces selection bias. Third, it focuses exclusively on RCTs, the gold standard for evaluating the efficacy of intervention measures, which ensures the high reliability of the research evidence included and lays a solid foundation for the validity of the meta-analysis results. Fourth, its methodology is rigorous in multiple aspects: sensitivity analyses are conducted to confirm the robustness of the results, preventing the impact of individual studies on the overall conclusion; a comprehensive outcome assessment is adopted, evaluating not only primary outcomes such as pain and distress but also secondary outcomes including crying time and maternal anxiety; in addition, validated scales are used for outcome measurement, such as WBFRS, NIPS, and FPS for assessing pain, as well as OSBD and OSBD-R for assessing distress, which provides a holistic view of the intervention effect and goes beyond the single-outcome focus of some previous studies. Fifth, the diverse sample characteristics enhance the external validity of the findings: the participants, ranging from 2 months to 6 years old (covering infants to preschoolers), are from 4 different countries, which demonstrates that music intervention can exert positive effects across different cultural contexts and developmental stages of children, making the research conclusions more applicable to a broader population.

Furthermore, the current analysis was limited to behavioral observation scales for pain and distress. The inclusion of physiological outcomes, such as heart rate variability (HRV), salivary cortisol levels, or skin conductance, would provide a more integrative and objective assessment of the stress response during vaccination. For instance, music interventions are theorized to modulate the autonomic nervous system, promoting parasympathetic activity which can be quantified by increased HRV (43). The concordance or discordance between behavioral scales (subjective/observed) and physiological biomarkers (objective) could offer deeper insights into the mechanism of action and strengthen the evidence base for music's efficacy. Future studies should aim to incorporate such multimodal assessment to capture the full spectrum of the intervention's impact.

However, limitations are also evident. First, the small number of included studies (5 RCTs) and total sample size (306 children) limits statistical power. This prevents subgroup analyses to explore factors like age (infants vs. preschoolers), music type (lullabies vs. children's songs), or intervention duration (5s pre-procedure vs. during vaccination), which could identify effectiveness-enhancing factors. Most critically, a *post-hoc* sensitivity analysis revealed that the statistically significant effect on pain was not robust to the exclusion of two studies for which data had to be extracted from figures. When these studies were removed, the effect was no longer significant, highlighting that the overall finding for pain is highly sensitive to the available data and methods of acquisition. This underscores an important limitation in the current evidence base and calls for caution in interpreting the pooled estimate for pain. The finding for distress was more stable in this regard. Second, publication bias cannot be formally assessed, funnel plots/Egger's test require ≥10 studies, so there is a risk of overestimating music's efficacy due to unpublished non-significant studies. Third, outcome measurement tools vary across studies—pain assessed via 4 scales (WBFRS, NIPS, FPS, FPS-R) and distress via 2 (OSBD, OSBD-R). Even with SMD adjusting for scale differences, varying psychometric properties (e.g., NIPS for infants vs. FPS-R for older children) may introduce heterogeneity. Fourth, some studies had unclear/high bias in allocation concealment and participant/personnel blinding, risking selection or performance bias, though objective measures (e.g., NIPS) mitigated this partially. Fifth, no long-term outcomes (e.g., needle phobia, future vaccination adherence) were reported in either document, leaving music's long-term impact unknown. Sixth, non-English studies were excluded, introducing potential language bias and limiting generalizability.

While this meta-analysis demonstrates the efficacy of music in reducing behavioral indicators of pain and distress, the absence of physiological outcome measures represents a limitation and an avenue for future research. The incorporation of objective biomarkers, such as heart rate variability (HRV), salivary cortisol levels, or galvanic skin response, would provide a more integrative biopsychosocial understanding of music's effects. For instance, an increase in HRV is a recognized indicator of parasympathetic nervous system activation and improved emotional regulation, which aligns with the proposed calming mechanism of music. The convergence—or intriguing divergence—between self-reported/observed scales and physiological data could offer deeper insights into the mechanisms of action, distinguishing between a mere masking of pain behaviors and a genuine attenuation of the underlying stress response. Future studies employing such multimodal assessment protocols are strongly encouraged to comprehensively capture the intervention's impact on the entire stress-pain axis.

## 5 Conclusions

This meta-analysis synthesized data from 5 RCTs to assess music intervention for pediatric vaccination pain and distress. Results showed music significantly reduced pain with sensitivity analyses confirming robustness. Overall, Music intervention may serve as a safe, low-cost non-pharmacological strategy, though further studies are needed to confirm these preliminary findings. Collectively, these findings suggest the potential for integrating music intervention into pediatric vaccination protocols to improve the patient experience.

## Data Availability

The original contributions presented in the study are included in the article/Supplementary Material, further inquiries can be directed to the corresponding authors.
