# Supplementary material for: Music intervention alleviates pain and distress in children undergoing vaccination: a systematic review and meta-analysis
Source: Front Pediatr. 2025 Nov 27;13:1699437. doi: 10.3389/fped.2025.1699437 (PMC12695783; doi:10.3389/fped.2025.1699437)
Supplement: Supplementary file 1 [file Supplementaryfile1.docx]

**Appendix S1:** Detailed Search Strategy

**Date of Search:** May 15, 2024
**Databases Searched:** PubMed, Embase (via Ovid), Cochrane Central Register of Controlled Trials (CENTRAL), Web of Science Core Collection
**Filters Applied:** None (to maximize sensitivity)
**Duplicate Removal:** Performed using EndNote 20, combining automatic and manual checking.

1. ****PubMed Search Strategy:****

("music therapy"[MeSH Terms] OR "music"[MeSH Terms] OR "music intervention*"[tiab] OR lullab*[tiab] OR "musical intervention*"[tiab]) AND

("child"[MeSH Terms] OR "child, preschool"[MeSH Terms] OR "infant"[MeSH Terms] OR child*[tiab] OR pediatric[tiab] OR paediatric[tiab] OR infant*[tiab]) AND

("vaccination"[MeSH Terms] OR "immunization"[MeSH Terms] OR vaccin*[tiab] OR immuniz*[tiab] OR immunis*[tiab] OR injection[tiab]) AND

("pain"[MeSH Terms] OR "stress, psychological"[MeSH Terms] OR pain[tiab] OR distress[tiab] OR fear[tiab] OR anxiety[tiab])

1. ****Embase (via Ovid) Search Strategy:****

1. exp music therapy/ or exp music/ or (music intervention* or lullab* or musical intervention*).ti,ab,kw.

2. exp child/ or exp preschool child/ or exp infant/ or (child* or pediatric or paediatric or infant*).ti,ab,kw.

3. exp vaccination/ or exp immunization/ or (vaccin* or immuniz* or immunis* or injection).ti,ab,kw.

4. exp pain/ or exp psychological stress/ or (pain or distress or fear or anxiety).ti,ab,kw.

5. 1 and 2 and 3 and 4

1. ****Cochrane Central Register of Controlled Trials (CENTRAL) Search Strategy:****

#1 (MeSH descriptor: [Music Therapy] explode all trees) OR (MeSH descriptor: [Music] explode all trees) OR ("music intervention*" OR lullab* OR "musical intervention*"):ti,ab,kw

#2 (MeSH descriptor: [Child] explode all trees) OR (MeSH descriptor: [Child, Preschool] explode all trees) OR (MeSH descriptor: [Infant] explode all trees) OR (child* OR pediatric OR paediatric OR infant*):ti,ab,kw

#3 (MeSH descriptor: [Vaccination] explode all trees) OR (MeSH descriptor: [Immunization] explode all trees) OR (vaccin* OR immuniz* OR immunis* OR injection):ti,ab,kw

#4 (MeSH descriptor: [Pain] explode all trees) OR (MeSH descriptor: [Stress, Psychological] explode all trees) OR (pain OR distress OR fear OR anxiety):ti,ab,kw

#5 #1 AND #2 AND #3 AND #4

1. ****Web of Science Core Collection Search Strategy:****

#1 TS=("music therapy" OR "music intervention*" OR lullab* OR "musical intervention*")

#2 TS=(child* OR pediatric OR paediatric OR infant*)

#3 TS=(vaccin* OR immuniz* OR immunis* OR injection)

#4 TS=(pain OR distress OR fear OR anxiety)

#5 #1 AND #2 AND #3 AND #4
