# Supplementary material for: Music intervention alleviates pain and distress in children undergoing vaccination: a systematic review and meta-analysis
Source: Front Pediatr. 2025 Nov 27;13:1699437. doi: 10.3389/fped.2025.1699437 (PMC12695783; doi:10.3389/fped.2025.1699437)
Supplement: Supplementary file 2 [file Supplementaryfile2.docx]

### ****Appendix S3. Outcome Measurement Instruments: Age Range and Psychometric Properties****

| **Instrument (Acronym)** | **Full Name / Description** | **Domain Measured** | **Validated Age Range / Target Population** | **Key Psychometric Properties (Examples)** | **Reference / Source** |
| --- | --- | --- | --- | --- | --- |
| ****Wong-Baker FACES® Pain Rating Scale (WBFRS)**** | A series of six faces ranging from a smiling face ("No hurt") to a crying face ("Hurts worst") | Self-reported Pain | **3 years and older** | Well-established validity and reliability in children for acute pain | Wong & Baker, 1988 |
| ****Neonatal Infant Pain Scale (NIPS)**** | A behavioral assessment tool with 6 items (facial expression, cry, breathing patterns, arm/leg movements, state of arousal) | Observed Pain | **Preterm and full-term neonates** (up to 1 year) | Valid and reliable for assessing procedural pain in infants; does not require verbal response | Lawrence et al., 1993 |
| ****Faces Pain Scale (FPS)**** | A series of seven faces depicting increasing levels of pain intensity | Self-reported Pain | **4 years and older** | Developed for children; shows good validity and reliability | Bieri et al., 1990 |
| ****Observational Scale of Behavioral Distress (OSBD)**** | An observational scale that rates the frequency and intensity of 11 operationally defined behaviors (e.g., cry, scream, restraint) | Observed Distress / Anxiety | **Children and adolescents** (typically 2-20 years in validation studies) | Validated for measuring distress during medical procedures; high inter-rater reliability | Jay et al., 1983 |
| ****Observational Scale of Behavioral Distress–Revised (OSBD-R)**** | A revised and updated version of the OSBD | Observed Distress / Anxiety | **Children and adolescents** | Improved behavioral anchors and scoring; maintains strong validity and reliability | Elliott et al., 1987 |
